# Supplementary material for: Comparative analysis of volume growth processes of Masson pine and Chinese fir forests in different regions of southern China
Source: PeerJ. 2025 Jan 6;13:e18706. doi: 10.7717/peerj.18706 (PMC11716076; doi:10.7717/peerj.18706)
Supplement: Supplemental Information 2 [file peerj-13-18706-s002.docx]

Description of the original data:

**national forest resource surveys** are from A report published by China Forestry Press in 2019. ISBN: 9787503899829

National Forestry and Grassland Administration. (2019). National forest resources statistics (2014-2018) (in Chinese). Beijing: *China Forestry Press.*

The document, titled mws_sm, contains three data tables, each representing distinct categories: Model Data, Data Processing, and Tabular Data. Below are the key variables included:

age: Represents the age of the trees (in years).

total: Denotes the total growth volume.

year: Refers to the annual growth increment.

mean: Indicates the average growth volume over a specified period.

rate: Represents the growth rate.

region: Categorizes the geographic regions, which include:

SC: South-Central region,

SE: Southeast region,

SW: Southwest region.

species: Identifies the tree species, which include Masson Pine (*Pinus massoniana*) and Chinese Fir (*Cunninghamia lanceolata*).

Science Data Centre and the National Earth System Data Centre

The DOIs for the datasets from **Science Data Centre and the National Earth System Data Centre** are as follows:

1. **China 30m resolution DEM Elevation, Slope, and Aspect dataset**: 10.12041/geodata.65449238360177.ver1.db
2. **China 1km resolution Annual Precipitation dataset**: 10.12041/geodata.113786088533256.ver1.db
3. **China 1km resolution Monthly Mean Temperature dataset**: 10.12041/geodata.164304785536614.ver1.db
4. **Annual average temperature data in China**10.12078/2022082501
5. **Boundary data of provincial administrative divisions in China for the year 2022**10.12078/2023010103
